# Evaluation of parameters extracted from tissue residue functions in dynamic susceptibility contrast MRI: Healthy volunteers examined during normal breathing and spontaneous hyperventilation

**DOI:** 10.1016/j.heliyon.2025.e42521

**Published:** 2025-02-06

**Authors:** Ronnie Wirestam, Arthur Chakwizira, Peter Reinstrup

**Affiliations:** a Dept. of Medical Radiation Physics, Lund University, Lund, Sweden; b Dept. of Intensive & Perioperative Care, Skåne University Hospital, Lund, Sweden

## Abstract

Dynamic susceptibility contrast magnetic resonance imaging (DSC-MRI) is the most common MRI method in clinical environments for assessment of perfusion-related parameters. In this study, special emphasis was placed on the shape of the tissue residue function under different physiological conditions. DSC-MRI-based parameters assumed to reflect arterial delay and cerebral oxygen extraction were obtained by deconvolution of tissue and arterial contrast-agent concentration time curves. The established mean transit time (MTT) estimate was supplemented by biophysical modelling for extraction of the oxygen extraction capacity, quantified in terms of an apparent oxygen extraction fraction (AOEF) index. Eight healthy volunteers were examined during normal breathing and spontaneous hyperventilation. Whole-brain MTT and AOEF increased during hyperventilation in all volunteers (average increase 33 % and 30 %, respectively). The arterial delay, reflecting the inverse of arterial flow rate, was also prolonged in all volunteers, and the mean arterial delay was 63 % longer during hyperventilation. The corresponding whole-brain MTT estimates were 3.8 ± 0.7 s during normal breathing and 5.0 ± 1.3 s during hyperventilation (mean ± SD, n = 8). The applied Bézier curve deconvolution algorithm returned tissue residue functions of plausible shapes, i.e., without oscillations and negative values, and some indications that curve shape is relevant for improved assessment of oxygen extraction properties were demonstrated.

## 1 Introduction

Dynamic susceptibility contrast magnetic resonance imaging (DSC-MRI) is the most common MRI method, in clinical environments, for assessment of perfusion-related parameters, typically including cerebral blood volume (CBV), cerebral blood flow (CBF) and mean transit time (MTT) [1,2,3]. Deconvolution of the tissue contrast agent (CA) concentration curve with the arterial input function (AIF) returns the tissue impulse response function (i.e., the tissue residue function scaled with the CBF). The tissue residue function is defined as the fraction of the amount of tracer that remains in the tissue of interest at a given time point after arrival. Conventionally, the height of the tissue impulse response function is used to estimate CBF, and, additionally, the area-to-height metric is frequently calculated to provide MTT [4,5]. CBV can be calculated as the time integral of the tissue CA concentration curve normalized to the time integral of the AIF, i.e., without the need for deconvolution [1].

Retrieval of additional information from the tissue residue function is indeed intriguing, and attempts have been made to extend the DSC-MRI data modelling and analysis to obtain parameters related to oxygen extraction during the passage of the blood through the local capillary network. For example, Østergaard et al. introduced the flow-heterogeneity concept [6], and the same group has later employed the capillary transit time heterogeneity (CTH or CTTH) parameter [7,8] and has also developed a comprehensive theoretical framework adapted to assessment of oxygen extraction capacity [7,8] or cerebral oxygen consumption [9].

A complicating factor is that common deconvolution algorithms, for example, those based on singular value decomposition (SVD) [3,10], tend to return oscillating tissue residue functions (including negative values) [11,12], which is in violation of the theoretical requirements that the tissue residue function must show non-negativity and strict decrease. Hence, recent approaches in the development of deconvolution algorithms [e.g., 12, 13, 14], including the control point interpolation (CPI) method by Mehndiratta et al. [14], have taken the ability to return physiologically reasonable tissue residue functions into account. In this study, deconvolution based on Bézier curves was applied [12]. The main advantage with a Bézier curve algorithm is that, in contrast to the CPI approach (in which the estimated residue function must pass through all control points) [14], a Bézier curve needs only to be bounded by the convex hull of its control points, and this markedly reduces the number of optimization parameters.

The oxygen extraction fraction (OEF) is defined as the fraction (or percentage) of oxygen that is removed from the blood by the tissue during the passage through the capillary network from the arterial to the venous side [15]. Extraction of parameters from the tissue residue function, or the associated transit time distribution, are unlikely to fully enable quantification of true OEF, even if the shape of the tissue residue function is accurately calculated [7]. However, DSC-MRI data in combination with careful biophysical modelling may contribute with comparable or supplementary parameters related to capillary function and tissue oxygenation [16,17]. In healthy volunteers at rest, a DSC-MRI-based OEF-related parameter, referred to as apparent OEF (AOEF), has previously shown significant, albeit moderate, correlation with conventional OEF, measured by quantitative susceptibility mapping (QSM) [18], and further experimental validation during a haemodynamic challenge would be a logical next step.

In the present study, experimental DSC-MRI data were processed, modelled and analysed to investigate whether parameters extracted from tissue residue curves were able to reflect physiological changes (such as decreased arterial flow rate and increased oxygen extraction) to be expected during controlled spontaneous hyperventilation in healthy volunteers. One issue of particular interest was to assess whether the AOEF metric provides additional value compared with the conventional MTT parameter in the ability to reflect oxygen extraction.

## 2 Material and methods

### 2.1 Subjects and experimental procedure

Eight healthy volunteers (male, mean age 33 years) were examined by DSC-MRI on two different occasions, once during normal breathing and once during hyperventilation. Spontaneous hyperventilation under external guidance was used to induce hypocapnia. Every other subject completed the experiment with normal breathing during the first visit. The time period between the two DSC-MRI experiments ranged from 3 to 35 days (mean 16.4 days). The volunteers adhered to their usual pattern of food and caffein intake during the days of examination. To reduce possible effects of diurnal variations in cerebral perfusion, both imaging sessions, for a given volunteer, were scheduled to start either in the morning or in the afternoon. In the MRI unit, the subjects were at rest in the supine position, with their eyes open and wearing earplugs. During the DSC-MRI experiments, end-tidal pCO_2_ (ETCO_2_) was monitored (see Table 1) and the subjects were breathing normal air with addition of extra O_2_ to a total level of 50 %, i.e., the fraction of inspired oxygen (FiO_2_) was 0.5.

**Table 1:** Age and ETCO_2_ level for each individual and the corresponding mean and standard deviation (SD) values [20]. *Note*: 1 kPa = *7*.50 mm *Hg* = *7.50* *Torr*.

**Volunteer no.**	**Age [years]**	**ETCO**_**2**_**[kPa]**	
		Normocapnia	Hypocapnia
1	40	6.0	4.6
2	39	5.6	4.1
3	35	5.0	3.7
4	31	5.7	2.7
5	31	5.8	3.1
6	30	5.8	4.4
7	30	5.6	4.0
8	29	5.3	3.6
Mean ± SD	33 ± 4.3	5.6 ± 0.32	3.8 ± 0.64

Nowadays, repeated injection of a gadolinium-based MRI contrast agent in healthy subjects is difficult to motivate, for ethical reasons, and the current study thus relies on a re-analysis of image data from a previously reported data collection protocol [19,20]. The present study is based on an independent scientific hypothesis and employs post-processing tools that were not available at the time of the previous publications. The experiments and subsequent post-processing were approved by the Ethical Review Board of the Faculty of Medicine, Lund University, Sweden. All procedures involving human participants were in accordance with the ethical standards of the institutional and/or national research committee and with the 1964 Helsinki declaration and its later amendments or comparable ethical standards. Written informed consent was obtained from each volunteer.

### 2.2 DSC-MRI experiment

DSC-MRI was carried out using a 1.5 T MRI whole-body unit (Siemens Magnetom Vision, Siemens Medical Systems, Erlangen, Germany). At each DSC-MRI experiment, 0.2 mmol/kg bodyweight of CA (Gadovist 1.0, Schering AG, Germany) was administered into a peripheral arm vein (injection rate 3 ml/s) followed by a saline flush. The CA passage through the brain was monitored during 75 s using dynamic gradient-echo echo-planar imaging (GRE-EPI) at a temporal resolution of 1.65 s. The imaging parameters were as follows: 10 slices, slice thickness 8 mm, echo time 54 ms, matrix size 128 × 128 and field of view 250×250 mm^2^. Subject-specific global AIFs were registered in middle cerebral artery branches in the Sylvian fissure region. Finally, in addition to standard DSC-MRI post-processing, signal-to-noise (SNR) levels in arterial and venous regions of interest (ROIs) were registered, using signal data from 11 baseline (i.e., pre-CA) images.

### 2.3 Post-processing and data analysis

The CA concentration estimate, calculated from the measured MRI signal S, is given by C_m_(t) = -ln[S(t)/S_0_]/(TE·r2∗), where r2∗ is the T2∗ relaxivity, t is time and S_0_ is the baseline signal. The general tracer-kinetic relationship in Eq. (1), where convolution is denoted “⊗”, was employed for quantification of perfusion parameters [2,3,5,12,21]:

(1) $${k_{H}C}_{m}\left(t\right)=CBF\left[R\left(t\right)⊗C_{a}\left(t-\delta \right)\right]$$

*CBF⋅R(t)*, obtained by deconvolution, is the tissue impulse response function and *R(t)* is the tissue residue function. The factor *k*_*H*_ is used to account for differences in haematocrit levels between large and small blood vessels (required for plasma tracers) and brain density [2]. The implementation of the Bézier deconvolution algorithm used in this study returns the bolus time delay *δ* between the site of the measured AIF and the local tissue inlet, i.e., the measured AIF is *C*_*a*_*(t-δ)*. Deconvolution was performed using cubic Bézier curves with the same constraints on the control points as described by Chakwizira et al. [12], which resulted in five free parameters for the estimation of *R(t)*. Thus, together with CBF and delay, the fitting involved a total of seven free parameters.

The mean transit time (MTT) was obtained according to Eq. (2) [5]:

(2) $$MTT=\int_{0}^{\infty }R\left(t\right)\text{dt}$$

The transit time distribution is given by *h(t) = -dR(t)/dt*, and the capillary transit time heterogeneity (CTH) can be calculated from the fitted residue functions *R(t)*, according to Eq. (3), following the discussion in the article by Mouridsen et al. [8].

(3) $$CTH=\sqrt{\int_{0}^{\infty }{\left(t-MTT\right)}^{2}\cdot h\left(t\right)dt}$$

Jespersen & Østergaard [7] and Mouridsen et al. [8] introduced the DSC-MRI-based metric oxygen extraction capacity (OEF_max_) *(*cf. Eq. (4)). In this report, we used the alternative term apparent OEF (AOEF), to emphasize that no attempts were made to interpret this parameter as true OEF in absolute terms:

(4) $$AOEF=\int_{0}^{\infty }h\left(\tau \right)Q\left(\tau \right)d\tau$$

where *h(τ)* is the capillary transit time distribution and *Q(τ)* is the oxygen extraction along a single capillary with transit time *τ*. AOEF is calculated as a sum of the oxygen extraction in a single capillary, over all transit times, weighted by the distribution of transit times over all capillaries. Assessment of the single-capillary oxygen extraction Q as a function of transit time τ requires appropriate modelling, and, in this study, the approach presented by Jespersen & Østergaard [7] is used, i.e., the capillary is treated as a three-compartment system with oxygen bound to haemoglobin, oxygen in plasma and oxygen in tissue. The transport of oxygen across the capillary membrane is assumed to be described by an exchange process with a fixed rate constant *k*. The rate of change in oxygen concentration along the capillary with a transit time τ is described by the differential equation in Eq. (5):

(5) $$\frac{dC}{dx}=-k\tau \left(\alpha_{H}P_{50}{\left(\frac{C}{B-C}\right)}^{1/h}-{\alpha_{H}\;pO}_{2}\right)$$

where C is oxygen concentration along the capillary, x is the normalized distance along the capillary, $\alpha_{H}=3.1\cdot 10^{-5}$ (mm Hg)^−1^ (Henry's constant), and B=0.1943 ml/ml (maximum amount of oxygen bound to haemoglobin). Furthermore, *h* is the Hill coefficient, $P_{50}$ is the oxygen pressure required for half saturation and *pO*_*2*_ is the tissue oxygen tension [7,8]. From the solution of Eq. (5) above, *Q* is given by *Q* = 1−C(1)/C(0) as a function of *kτ*, enabling calculation of AOEF.

Breathing of air with added oxygen to a total O_2_ level of 50 % is likely to influence OEF [15], and it will also increase *pO*_*2*_, compared with breathing of normal air [22]. In addition, hyperventilation will reduce *P*_*50*_ and *h* [23], and lower *pO*_*2*_ [24,25], compared with normal breathing. The values used in the current application of the model are given in Table 2. The rate constant *k* is usually set to yield an AOEF of 30 % in healthy white matter [8]. However, considering that OEF is likely to have deviated from the normal value also in the case of normal breathing rate (due to the extra O_2_), we refrained from using a pre-defined white-matter oxygen extraction value of 30 % to determine *k*. Instead, we used the literature value of *k* = 118 s^−1^, proposed by Jespersen & Østergaard [7], and made no presumptions about AOEF, for normalization purposes, neither at normal breathing nor during hyperventilation.

**Table 2:** Values of *k*, *P*_*50*_, *h* and *pO*_*2*_ assumed in the application of the biophysical model for normal breathing and hyperventilation at the given fraction of inspired oxygen (FiO_2_).

	Normal breathing *FiO*_*2*_ = 0.5	Hyperventilation *FiO*_*2*_ = 0.5
*k*	118 s^−1^	118 s^−1^
*P*_*50*_	26 mm Hg	24 mm Hg
*h*	2.8	2.6
*pO*_*2*_	32 mm Hg	28 mm Hg

All parameters were calculated pixel by pixel. After segmentation of brain parenchyma using ITK-SNAP (version 3.8.0, www.itksnap.org) [26], MTT and AOEF estimates were extracted as the mean parameter value of all non-zero pixel values in the segmented brain tissue volume. For the delay *δ*, outliers (defined as values more than three scaled median absolute deviations from the median) were removed prior to calculation of the mean parameter value of all remaining non-zero pixel values in the segmented brain tissue volume.

The available image data did not enable calculation of other types of MRI parameters used to assess venous oxygen saturation or OEF, for example, T2∗ or phase/QSM. The fact that T2∗ has been shown to be closely related to haemoglobin oxygen saturation implies that the venous GRE signal must decrease with lowered oxygen saturation level [27], i.e., the venous signal must decrease with increasing OEF. Hence, the registered venous SNR values were compared between normal breathing and hyperventilation, in each subject, serving as a very crude substitute for T2∗ to monitor potential alterations in whole-brain OEF caused by hyperventilation. SNR was also recorded in a brain-feeding artery to ensure that no systematic MRI-system-related change in SNR had occurred between the two different measurement occasions. Changes in observed venous SNR between hyperventilation (H) and normal breathing (N) were calculated in terms of the ratio SNR_H_/SNR_N_ and compared with the corresponding changes in MTT, AOEF, δ and CTH. Extracted MRI parameters were also related to measured ETCO_2_ levels.

### 2.4 Statistical analysis

For all statistical analyses, the level of significance was α = 0.05. Prior to hypothesis testing, a Shapiro-Wilk test was used to test for normal distribution. A paired *t*-test was applied to determine whether the AOEF, MTT and venous SNR values observed during normal breathing were significantly different from those observed during hyperventilation. For delay data, the hypothesis of normal distribution had to be rejected, and a Wilcoxon test was instead used to test for significant difference in *δ* between normal breathing and hyperventilation. The issue of family-wise error rates for multiple hypothesis tests was addressed using the Bonferroni method, i.e., obtained p-values were compared with α/4 = 0.0125. The MTT-versus-ETCO_2_, AOEF-versus-ETCO_2_, SNR-versus-ETCO_2_, δ-versus-ETCO_2_, CTH-versus-ETCO_2_, MTT_H_/MTT_N_-versus-SNR_H_/SNR_N_, AOEF_H_/AOEF_N_-versus-SNR_H_/SNR_N_, *δ*_H_/δ_N_-versus-SNR_H_/SNR_N_, CTH_H_/CTH_N_-versus-SNR_H_/SNR_N_ and CTH_H_/CTH_N_-versus-AOEF_H_/AOEF_N_ relationships were evaluated by linear-regression and correlation analyses.

## 3 Results

In all 8 volunteers, the DSC-MRI-based estimates of MTT and AOEF increased when the subject was hyperventilating (Table 3), and the average increases during hyperventilation were 33 % and 30 %, respectively. Additionally, in all 8 subjects, a lower venous baseline SNR was observed during hyperventilation (Table 3), with a mean SNR decrease of 28 % compared with normal breathing. Hyperventilation did not cause any systematic SNR alteration in the observed arterial ROIs (i.e., some volunteers exhibited a slight arterial SNR increase while others showed a slight decrease, mean change +6 %).

**Table 3:** Individual MTT, AOEF, venous SNR and arterial delay estimates obtained during normal breathing (N) and hyperventilation (H) together with the corresponding mean and SD values.

	**MTT [s]**		**AOEF [a.u.]**		**Venous SNR [a.u.]**		**Delay δ [s]**	
Volunteer #	N	H	N	H	N	H	N	H
1	2.98	5.83	20.3	32.6	48.2	22.5	0.132	1.00
2	3.16	3.69	22.5	28.7	34.0	27.2	0.489	0.608
3	4.68	6.27	27.1	31.1	32.1	25.6	0.879	1.01
4	3.39	4.11	23.2	26.4	24.8	22.2	0.396	0.541
5	3.52	3.76	19.8	26.6	35.9	24.9	0.253	0.638
6	5.05	6.97	22.3	30.6	33.4	25.2	0.696	0.972
7	3.33	4.04	22.7	26.9	31.2	25.4	0.571	0.737
8	3.93	5.26	22.4	29.1	36.3	20.4	0.530	0.935
Mean	3.76	4.99	22.5	29.0	34.5	24.2	0.493	0.805
SD	0.75	1.3	2.2	2.3	6.6	2.2	0.24	0.19

The bolus time delay *δ* between the site of the measured AIF (i.e., the Sylvian fissure region) and the local tissue inlet was longer during hyperventilation in all 8 volunteers (Table 3), implying lower arterial blood velocity during hyperventilation. The mean arterial delay was 63 % longer during hyperventilation, corresponding to a 39 % reduction in arterial blood velocity if the distance travelled was the same.

The statistical *t*-test analysis showed that MTT, AOEF and SNR estimates differed significantly between normal-breathing conditions and hyperventilation (p = 0.0049, p = 0.011 and p = 0.0055, respectively). The Wilcoxon test indicated that δ was significantly longer during hyperventilation than during normal breathing (p = 0.0078). In all cases, the null hypothesis was rejected while controlling the family-wise error rate at level α = 0.05.

**Fig. 1:**
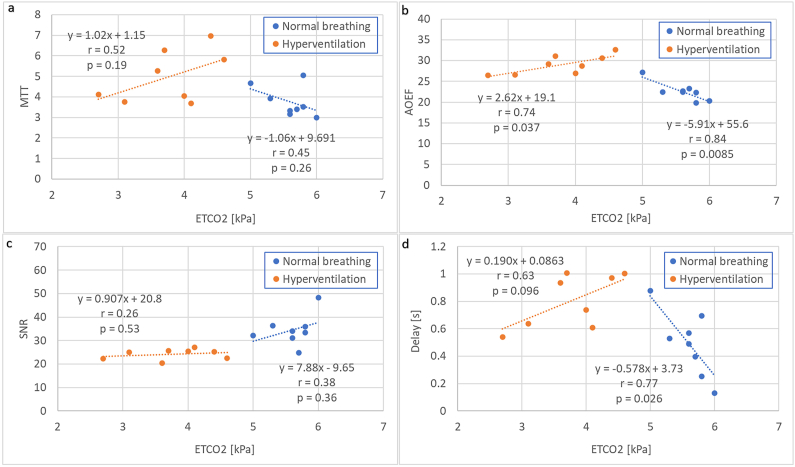
Estimates of (a) mean transit time (MTT), (b) apparent oxygen extraction fraction (AOEF), (c) venous signal-to-noise ratio (SNR) and (d) arterial delay (δ) obtained during normal breathing and hyperventilation as a function of end-tidal pCO_2_ (ETCO_2_).

**Fig. 2:**
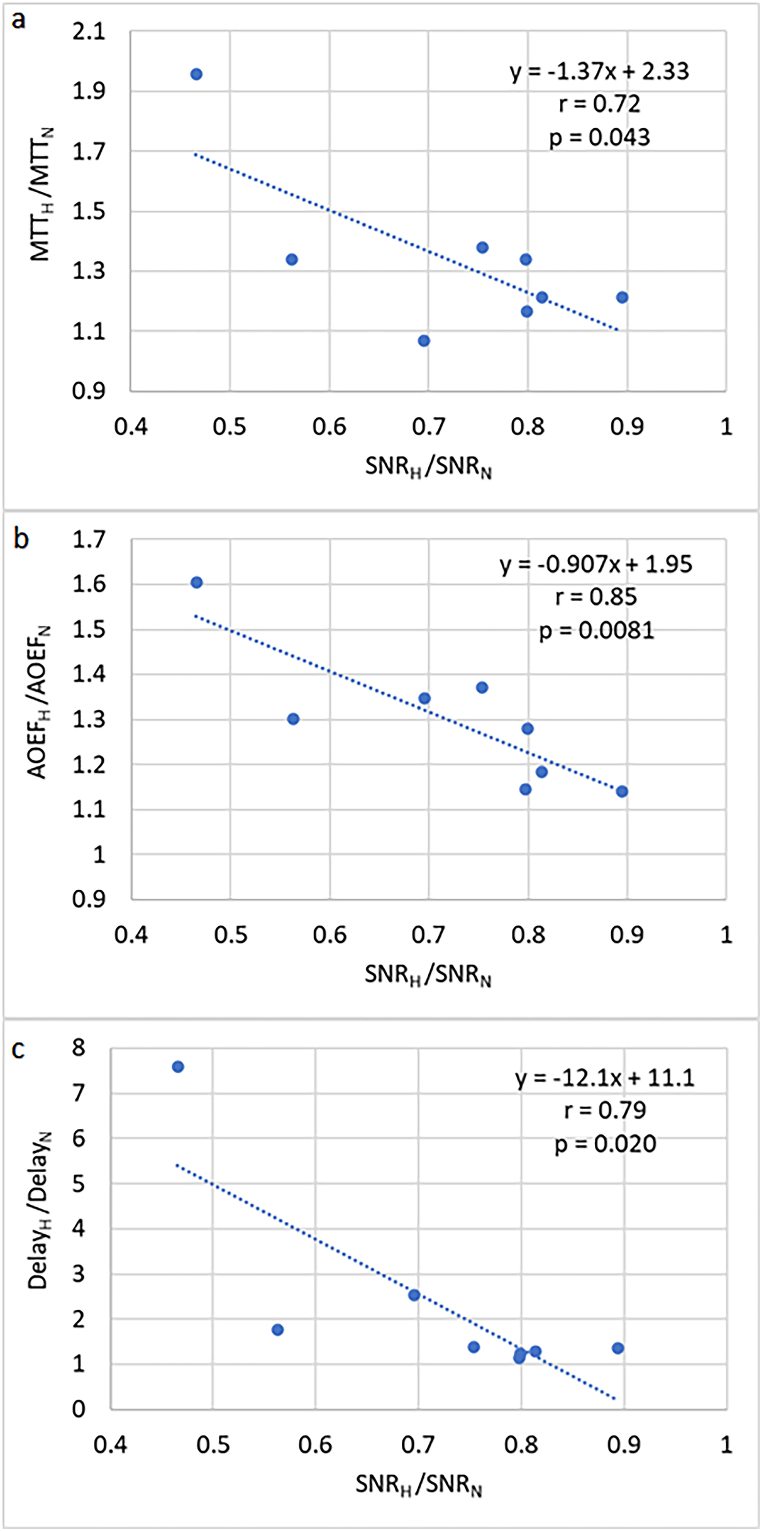
Observed relationships of how the change in MTT, the change in AOEF and the change in arterial delay, between normal breathing (N) and hyperventilation (H), depended on the corresponding change in venous SNR, i.e. (a) MTT_H_/MTT_N_ versus SNR_H_/SNR_N_, (b) AOEF_H_/AOEF_N_ versus SNR_H_/SNR_N_ and (c) *δ*_H_/δ_N_ versus SNR_H_/SNR_N_.

The observed associations between MTT and ETCO_2_, AOEF and ETCO_2_, SNR and ETCO_2_ and δ and ETCO_2_ are shown in Fig. 1(a–d), while MTT_H_/MTT_N_-versus-SNR_H_/SNR_N_, AOEF_H_/AOEF_N_-versus-SNR_H_/SNR_N_ and *δ*_H_/δ_N_-versus-SNR_H_/SNR_N_ relationships are displayed in Fig. 2(a–c). For completeness, CTH_H_/CTH_N_-versus-SNR_H_/SNR_N_, CTH_H_/CTH_N_-versus-AOEF_H_/AOEF_N_ and CTH-versus-ETCO_2_ relationships are available in Supplementary Fig. S1.

**Fig. 3:**
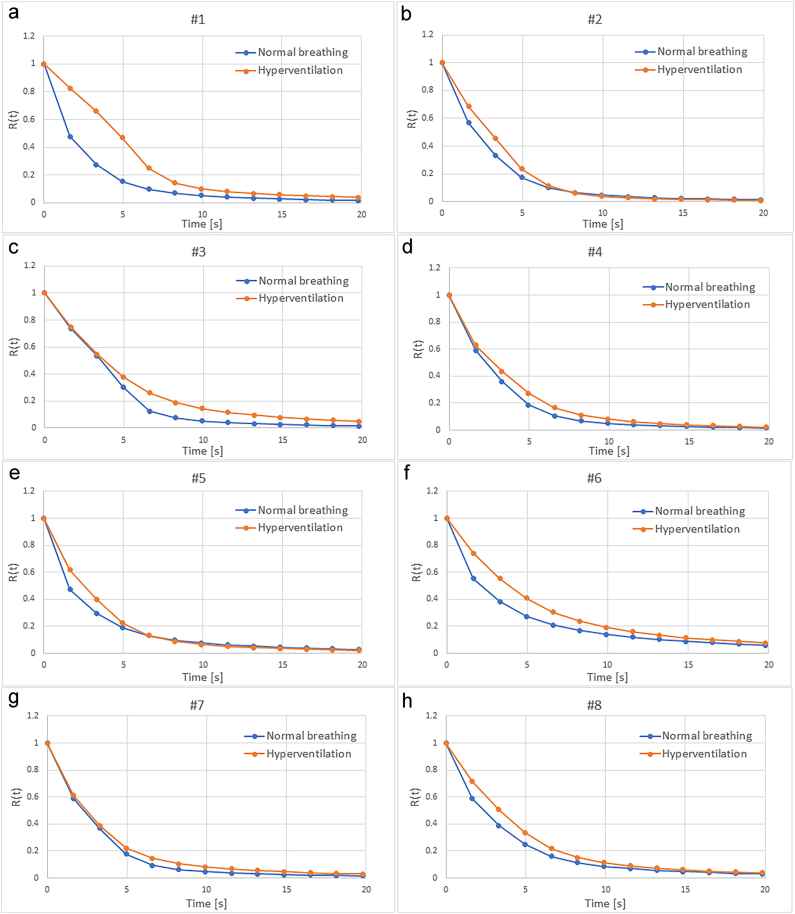
Examples of tissue residue functions R(t) returned by the Bézier deconvolution algorithm. (a–h) Tissue residue functions R(t) from volunteers #1 to #8. The R(t) curves shown represent mean values from a slice located just above the lateral ventricles.

Examples of tissue residue functions R(t), returned by the Bézier deconvolution algorithm, are displayed in Fig. 3(a–h) for volunteers #1 to #8. The R(t) curves shown represent mean values from a slice located just above the lateral ventricles.

## 4 Discussion

In previously reported analyses, the volunteers included in this study showed a CBF decrease of 29 % and a CBV decrease of 17 % during hyperventilation [19,20], in good agreement with previous studies [28,29,30]. Moderate hyperventilation has been shown to cause decreased CBF and increased cerebral oxygen extraction [31,32], and this is normally assumed to result in a more or less sustained cerebral oxygen metabolism [32]. This expected increase in OEF is in overall agreement with the observations made in the present study, i.e., the decreased venous SNR, the prolonged MTT, and the increased AOEF, all seem to be intuitively consistent with an increased level of oxygen extraction. It is particularly promising that the expected increase in MTT and AOEF during hyperventilation could be observed in all volunteers and not only on group level.

In correspondence with the well-established decrease in global CBF, hypocapnia causes a decrease in the blood velocity in major brain-feeding arteries. Ide et al. presented an exponential relationship between relative peak velocity in the middle cerebral artery (MCA) and the change in end-tidal pO_2_ (ΔETCO_2_) [33]. This relationship predicts that the ΔETCO_2_ registered in the present study, i.e., ΔETCO_2_ = −1.8 kPa = −13.5 Torr (*cf*. Table 1), would result in a decrease in arterial peak velocity of 30 %, and this is in reasonable agreement with the 39 % decrease that corresponds to the observed change in mean arterial delay in the present study. The bolus time delay estimates tended to show some voxels with spuriously high *δ* values within the brain volume, and this might have influenced the mean values in some volunteers. The median value is less sensitive to such effects, and the median value of the relative change in arterial delay indicated that the arterial delay was 35 % longer during hyperventilation, corresponding to a 28 % reduction in arterial blood velocity if the distance travelled was the same. Hence, even if the arterial delay parameter may have been slightly less robust than the other parameters evaluated in this study, the overall impression, considering both mean and median values, is that the Bézier algorithm performs reasonably well also in this respect.

On group level, all tested parameters showed values during hyperventilation that were significantly different from those seen during normal breathing conditions (p < α/4), and the observed average increase in MTT of 33 % is in excellent agreement with Fortune et al. [28] who observed a decrease of 7.2 % for CBV and 30.7 % for CBF, implying a change in MTT with a factor of 0.928/0.693 = 1.34, according to the central volume theorem (MTT = CBV/CBF). Similarly, Ito et al. [29] reported a prolongation of MTT by 44 % during hypocapnia, also in quite reasonable agreement with the results of the present study. In absolute terms, the global MTT estimate observed in the present study (mean value 3.8 s at normoventilation) was comparable to other studies [29,34,35], although observations of somewhat longer values have also been reported [36,37,38]. It should, however, be noted that conventional SVD-based deconvolution algorithms tend to show a risk, at least at certain regularization settings [39], of underestimating the peak value of the tissue impulse response function [12], and some previous DSC-MRI investigations may thus have arrived at correspondingly overestimated MTT levels.

The validity of the observed change in AOEF is more difficult to assess, partly because the subjects were breathing O_2_ to a total fraction of 50 %. A crude attempt was made to account for this in the biophysical model (Table 2), by using reasonable model input values, but no attempts were made to calibrate the model to return absolute levels of OEF or oxygen extraction capacity in this study. It should be noted that an elevated FiO_2_ can be expected to result in a reduction of OEF [15], so observations of AOEF values that are lower than the oxygen extraction capacity (OEF_max_) values previously stated for controls, by Jespersen & Østergaard [7], is not unreasonable. During moderate hyperventilation, it is normally, as pointed out above, assumed that the cerebral metabolic rate of oxygen (CMRO_2_) is kept relatively unchanged, although indications of a slight decrease (less than 5 %) have recently been reported [40]. Since CBF can be assumed to decrease by approximately 30 %, a simultaneous decrease in CMRO_2_ limited to, for example, 4 % would require OEF to increase by a factor 0.96/0.7 = 1.37, which is reasonably close to the observed mean AOEF increase of 30 % in the present study.

MTT has previously been shown to correlate with OEF [18,41,42,43], which is logical considering that a longer residence time of blood within the tissue segment allows for more oxygen to be delivered to the tissue. In the microvasculature modelling study by Park & Payne [41], it was pointed out that a prolongation of MTT, resulting from a decrease in CBF, will cause an increase in OEF, as there is more time for oxygen to be extracted in order to preserve CMRO_2_. The AOEF parameter, corresponding to the oxygen extraction capacity quantity introduced by Jespersen & Østergaard [7], is less established than MTT, and one aim of this study was to provide further indications of whether or not AOEF provides additional value compared with MTT in the ability to reflect oxygen extraction. The established OEF parameter, measured by positron emission tomography (PET), has previously exhibited a strong inverse correlation with ETCO_2_ in healthy volunteers [44], and the association with ETCO_2_ was thus investigated for all parameters assumed to be affected by hyperventilation in this study. SNR, MTT, arterial delay and AOEF showed weak to very strong correlations with ETCO_2_ during normal breathing, and it was indeed promising that the highest coefficient of correlation was observed for AOEF (r = 0.84, p = 0.0085). Interestingly, the correlation with ETCO_2_ was lost during hyperventilation (below approximately 4.5 kPa) in all cases (cf. Fig. 1(a–d)). The relationship between hyperventilation and CBF is complex [45], but several previous studies have reported that the decreases in CBF and in cerebral blood flow velocity tend to cease when ETCO_2_ goes below approximately 35 mm Hg (4.7 kPa) [46,47,48], and when the CBF level is stabilized it would be reasonable to expect no further increase in OEF with decreased ETCO_2_. This supports the current findings, and, with regard to the robustness of the current analysis, it is encouraging to note that not only the parameters derived from the bolus passage through the capillary network (i.e., MTT and AOEF) showed this trend, but also the delay (related to arterial bolus transport) and the SNR measurement (which is entirely independent of the CA administration) behaved similarly.

Another interesting analysis, assuming that venous SNR very roughly would inversely reflect the whole-brain OEF level, was to investigate the association between the relative hyperventilation-induced changes in MTT and AOEF and the corresponding relative hyperventilation-induced change in venous SNR. Both MTT and AOEF changes showed significant correlations with venous SNR change, and, also in this case, AOEF showed a higher correlation coefficient than MTT (r = 0.85 versus r = 0.72), potentially indicating that the AOEF metric shows a higher sensitivity to changes in oxygen extraction than MTT. Arterial delay *δ* is inversely related to flow, and a correspondingly expected negative correlation between delay change and SNR change was observed (r = 0.79, p = 0.02), strengthening the assumption that venous SNR does, in fact, reflect the venous oxygen saturation level. The change in CTH, however, did not correlate either with SNR change or with AOEF change (Figs. S1(a–b)). These observations appear not to contradict previous predictions (see Fig. 3A in Ref. [49]), in the sense that the simulations by Østergaard et al. indicated that when CBF decreases, the associated increase in OEF is not conditional on a change in CTH, i.e., at low CBF, high OEF levels can be achieved for all degrees of CTH [49]. Similarly, CTH did not correlate with ETCO_2_ (Fig. S1(c)), and this observation does not seem to be in immediate conflict with the introductory statement of Vogel & Kuschinsky in Ref. [50] that “[w]hen CBF was decreased by hypocapnic hyperventilation, the plasma perfusion heterogeneity changed incoherently” [50,51].

A crucial issue when applying DSC-MRI for assessment of an oxygen extraction index is the quality of the tissue residue function, retrieved by deconvolution of the tissue concentration curve with the arterial input function. From a theoretical viewpoint, the Bézier-curve-based deconvolution algorithm, employed in this study, possesses several advantages in this context, for example, by providing tissue residue functions that are model-independent, strictly decreasing and free from oscillations of the type that typically perturb the output from SVD-based algorithms. Hence, it is of interest to compare the quantitative results of this study with visual inspections of corresponding tissue residue functions, obtained during normal breathing and hyperventilation, in order to gain further insight into the potential advantages of using the full shape of the tissue residue function, as in the AOEF calculation, compared with using only a summary metric, i.e. the area under curve, for MTT estimation (Eq. (2)). When comparing two physiological situations, in this case normal breathing and hyperventilation, it is likely that AOEF would offer limited advantages over MTT if the tissue residue functions from both situations were to belong to the same class or type of shapes. Looking at the qualitative examples in Fig. 3, volunteers #1 (Fig. 3(a)), #2 (Fig. 3(b)), and #5 (Fig. 3(e)), and possibly also #3 (Fig. 3(c)), show curve shapes that are inherently different between normal breathing and hyperventilation. For #2 and #5, the two curves show a subtle crossing point, i.e., values during hyperventilation were higher than during normoventilation at early time points but lower than normoventilation at later time points. For #3, the initial part of the normoventilation curve appears to be linear rather than exponential. For #1, the hyperventilation curve is markedly different in shape, exhibiting a less exponential and more sigmoid shape. For the remaining volunteers, curve shapes are considerably more similar when comparing normal breathing and hyperventilation (cf. Fig. 3(d, f, g, h)). Correspondingly, it can be noted that volunteers #1, #2, #3 and #5 are, in fact, the ones that show the largest absolute differences between the MTT_H_-to-MTT_N_ ratio and the AOEF_H_-to-AOEF_N_ ratio. Assuming that the venous SNR ratio can be used as a bolus-tracking-independent measure of OEF change, it is even more interesting to note that the two data points in Fig. 2(a) that deviate the most from the linear regression line correspond to #1 and #5, indicating that using a parameter based on the shape of the residue curve, instead of using MTT, may be of relevance in establishing a DSC-MRI metric that more precisely reflects OEF. It is also of interest to note that volunteer #1 exhibited the highest venous SNR drop during hyperventilation, as well as the largest change in arterial delay time δ, while at the same time showing the highest degree of deviating curve shape during hyperventilation. In a previous analysis of the same group of volunteers [20], which allowed for improved precision in absolute CBF estimates, it was reported that volunteer #1 did, in fact, show the highest change in CBF during hyperventilation (54 % decrease), an observation that is consistent with the largest change in arterial delay time seen in the present analysis. Hence, the current results show some indications that physiological challenges that prompt large changes in OEF, in order for cerebral oxygen metabolism to be maintained, are indeed reflected by observable changes in the *R(t)* shape, and that metrics that make use of the entire *R(t)* curve (not only the time integral) may provide additional value in DSC-MRI-based assessments of oxygen extraction.

The biophysical model relies on literature values of *h*, $P_{50}$ and *pO*_*2*_ and, compared to the studies by Jespersen & Østergaard [7] and Mouridsen et al. [8], *h* and $P_{50}$ had to be modified to account for hyperventilation, while *pO*_*2*_ was also adjusted to take the effects of O_2_ inhalation into consideration. For cerebral capillaries, Dahl et al. [23] reported approximately 8 % decrease in $P_{50}$ and a corresponding reduction in *h* by approximately 8 %, during hyperventilation, and we applied the same relative changes in our study. With regard to *pO*_*2*_, Nielsen [22] stated in a well-established textbook that, in an ‘oxygen challenge test’, one should ensure that a *pO*_*2*_ increase of >20 mm Hg is recorded when increasing FiO_2_ to 1.0. A simple rescaling of this 20 mm Hg increase implies that a minimum increase of 7 mm Hg would be expected when FiO_2_ is increased from 0.21 to 0.5, and we therefore employed *pO*_*2*_ = 32 mm Hg at normal breathing in this study. Finally, we assumed that hyperventilation reduced *pO*_*2*_ by approximately 11–12 % [24,25] leading to a *pO*_*2*_ level of 28 mm Hg during hyperventilation.

A study design relying on a re-analysis of existing experimental data has inherent drawbacks, for example, in terms of the inability to acquire complementary data or to use updated measurement protocol settings or benefit from improved MRI scanner properties. Another potential limitation in this case was the rather small number of volunteers, but the effect size must also be taken into account when addressing the sample size issue. Considering that this investigation was based on a re-analysis of previously acquired data, it was not meaningful to perform any formal sample-size calculation in this study. However, in a previous investigation [20], based on the same dataset as in the present study, the mean CBF difference between pairs (normal breathing versus hyperventilation) was 21.0 ml/(min 100 g) and the SD of these differences was 16.0 ml/(min 100 g), and this would imply a sample size of 7 (paired *t*-test, power 0.8 and α = 0.05). Although CBF estimation was not part of the present study, the size of the CBF changes is closely associated with alterations in arterial velocity (reflected by δ) and oxygen extraction during hyperventilation. Another source of error is that literature values of *h*, $P_{50}$ and *pO*_*2*_ are needed as input to the biophysical model. The employed literature values that corresponded to modified conditions, in comparison with normal breathing of air, were selected to be representative estimates, introduced to demonstrate that the parameters returned by the biophysical model are reasonably robust to physiological challenges. The assigned literature values do not, however, represent mean values extracted from a complete inventory of the current literature. Hence, the AOEF parameter should not be overinterpreted in terms of absolute quantification.

In conclusion, DSC-MRI in combination with Bézier deconvolution showed promising results in the detection of changes in cerebral oxygen extraction, induced by spontaneous hyperventilation, in healthy subjects. All subjects displayed increased MTT, increased AOEF, decreased venous SNR and increased arterial delay during hyperventilation, in accordance with expectations, and a previously reported correlation between PET-based OEF and ETCO_2_, under resting conditions, was reproduced for AOEF. Several observations indicated that access to complete tissue residue curves of high quality may enable better oxygen extraction characterization than using only MTT, in particular when OEF changes are believed to be substantial.

## CRediT authorship contribution statement

**Ronnie Wirestam:** Writing – original draft, Visualization, Software, Methodology, Investigation, Funding acquisition, Formal analysis, Data curation, Conceptualization. **Arthur Chakwizira:** Writing – review & editing, Software, Methodology. **Peter Reinstrup:** Writing – review & editing, Methodology, Investigation.

## Ethics statement

The experiments were approved by the Ethical Review Board of the Faculty of Medicine, Lund University, Sweden, with the approval number LU 527–98. All participants provided written informed consent to participate in the study and for their data to be published.

Funding

This study was financially supported by the Swedish Research Council (Grant No. 2017-00995).

## Data and code availability statement

Data will be made available on request. For requesting data, please contact the corresponding author.

## Declaration of competing interest

The authors declare the following financial interests/personal relationships which may be considered as potential competing interests:Ronnie Wirestam reports financial support was provided by Swedish Research Council. If there are other authors, they declare that they have no known competing financial interests or personal relationships that could have appeared to influence the work reported in this paper.
