# Supplementary material for: Evaluation of parameters extracted from tissue residue functions in dynamic susceptibility contrast MRI: Healthy volunteers examined during normal breathing and spontaneous hyperventilation
Source: Heliyon. 2025 Feb 6;11(4):e42521. doi: 10.1016/j.heliyon.2025.e42521 (PMC11867289; doi:10.1016/j.heliyon.2025.e42521)
Supplement: Multimedia component 1 [file mmc1.pdf]

Supplementary Figure S1

a

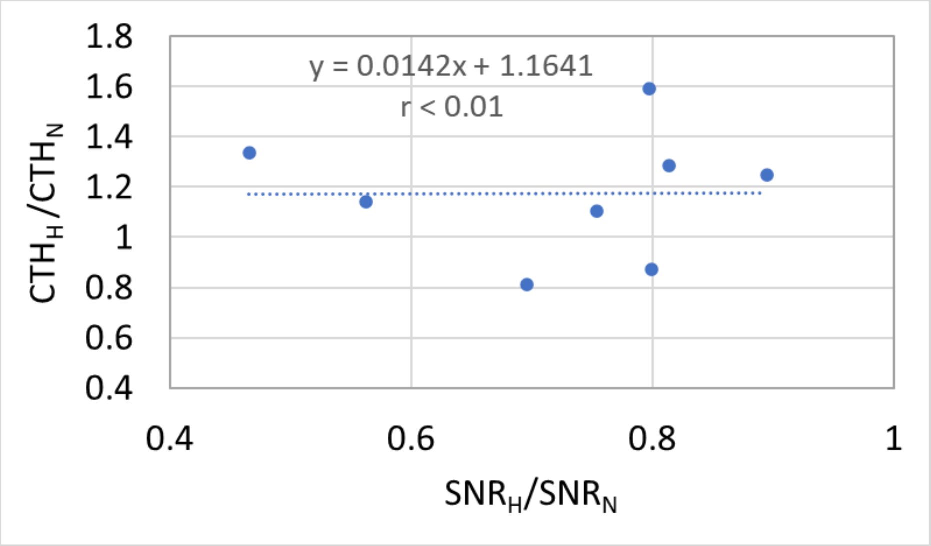

b

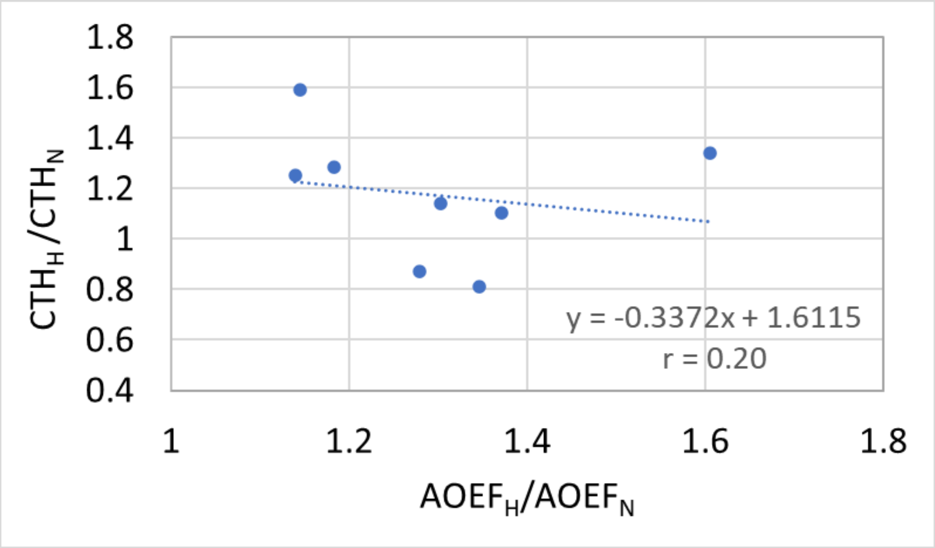

c

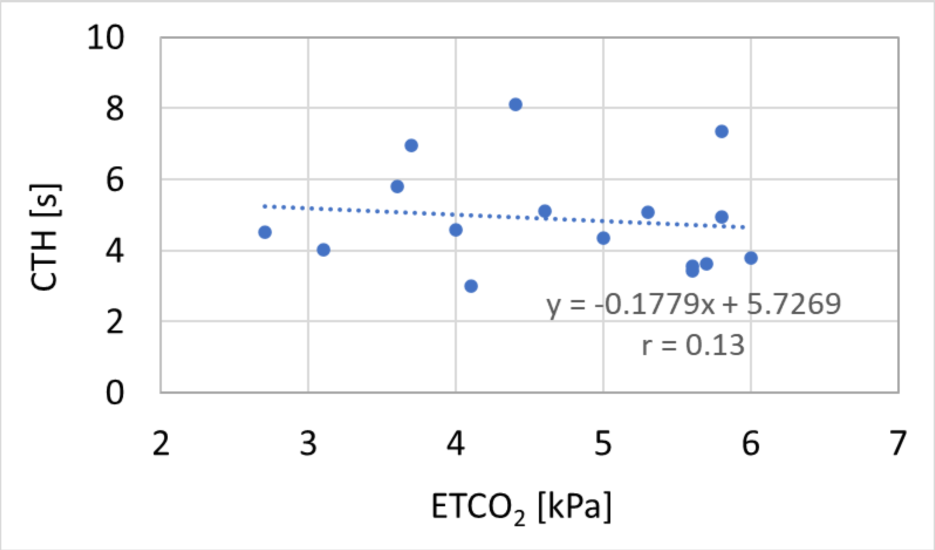

Figure S1 (a) CTH_H/CTH_N versus SNR_H/SNR_N. (b) CTH_H/CTH_N versus $AOEF_H/AOEF_N$. (c) CTH as a function of $ETCO_2$. CTH – Capillary transit time heterogeneity, SNR – venous signal-to-noise ratio, AOEF – apparent oxygen extraction fraction, $ETCO_2$ – End-tidal pCO_2, H – hyperventilation, N – normal breathing.
